# P066: Risk factors of vancomycin-resistant enterococcus colonization in hemologic patients

**DOI:** 10.1186/2047-2994-2-S1-P66

**Published:** 2013-06-20

**Authors:** V Mioljevic, L Markovic-Denic, A Vidovic, D Tomin

**Affiliations:** 1 Department of Hospital Epidemiology and Hygiene, Clinical Center of Serbia, Serbia; 2 Institute of Epidemiology, Faculty of Medicine, University of Belgrade, Serbia; 3 Clinic of Hematology, Clinical Center of Serbia, Serbia; 4 Faculty of Medicine, University of Belgrade, Belgrade, Serbia; 5 Clinic of Hematology, Faculty of Medicine, University of Belgrade, Belgrade, Serbia

## Introduction

Vancomycin-resistant Enterococci (VRE) is one of the most important hospital pathogens.

## Objectives

The aim of the study was to evaluate the VRE colonization in patients hospitalized at the Hematology Intensive Care Unit and associated risk factors.

## Methods

Prospective cohort study involved 70 patients hospitalized at the Intensive Care Unit (ICU), Clinic for Hematology, during three months. Demographic data and data risk factors for VRE colonization during present and previous hospitalization (within 6 months) were recorded for each patient using the questionnaire. Feces or rectal swab was collected for culture from patients on admission and at discharge in case when VRE was not isolated on admission. The *Enterococci* were isolated by standard microbiological methods. Isolate sensitivity was tested by disk-diffusion test using the 30μg/ml (BBL) Vancomycin plates according to CLSI standard.

## Results

Upon admission, 7% of patients were already colonized with VRE. The rate of VRE colonization during present hospitalization was 41.5%. Univariate logistic regression demonstrated statistical significant differences of acute myeloid leukemia (AML) diagnosis (RR=3.1; 95%CI 1.1-8.6; p=0.03), length of present stay (RR=1.1; 95%CI 1.1-1.2; p=0.002), use of aminoglycosides (RR=3.9; 95%CI 1.1-13.1; p=0.03), and pip/tazobactam (RR=4.7; 95%CI 1.6-13.9; p=0.005) in present hospitalization, duration of use of carbapenem (RR=1.2; 95%CI 1.1-1.3; p=0.05) and pip/tazobactam (RR=1.4; 95%CI 1.3-1.7; p=0.006) in present hospitalization between the VRE-colonized and non-colonized patients. AML, use of carbapenem in previous hospitalization and duration of use of piperacillin/tazobactam in present hospitalization were independent risk factors of VRE-colonized patients according to multivariate logistic regression.

## Conclusion

VRE colonization rate was high among patients admitted to hematology ICU. Rational use of antibiotics and an active surveillance may be helpful preventive measures for development of bacterial resistance to antimicrobial agents.

## Disclosure of interest

None declared

